# *N*,*N*-Dimethyl-*N*′,*N*′′-diphenyl­phospho­ric triamide

**DOI:** 10.1107/S1600536811046058

**Published:** 2011-11-09

**Authors:** Mehrdad Pourayoubi, Mohammad Yousefi, Farnaz Eslami, Arnold L. Rheingold, Chao Chen

**Affiliations:** a Department of Chemistry, Ferdowsi University of Mashhad, Mashhad, 91779, Iran; b Department of Chemistry, Shahr-e Rey Branch, Islamic Azad University, Tehran, Iran; c Department of Chemistry, University of California, San Diego, 9500 Gilman Drive, La Jolla, CA 92093, USA

## Abstract

In the title compound, C_14_H_18_N_3_OP, a crystallographic mirror plane bis­ects the mol­ecule (the C,N,C atoms of the dimethyl­amido moiety and the P=O unit lie on the mirror plane). The P atom has a distorted tetra­hedral geometry; the bond angles at P are in the range 98.98 (11)–115.28 (7)°. In the crystal, the O atom of the P=O group acts as a double hydrogen-bond acceptor for two symmetry-equivalent N—H⋯O hydrogen bonds, building [001] chains containing *R*_2_^1^(6) loops.

## Related literature

For bond lengths and angles in compounds having a [(N)P(O)(N)_2_] skeleton, see: Sabbaghi *et al.* (2011 ▶). For the double hydrogen-bond acceptor capability of the phosphoryl group, see: Pourayoubi *et al.* (2011 ▶).
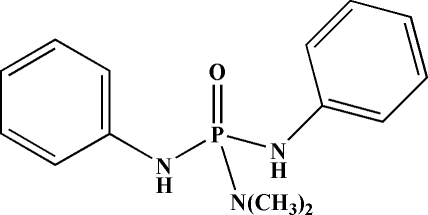


## Experimental

### 

#### Crystal data

C_14_H_18_N_3_OP
*M* *_r_* = 275.28
Orthorhombic, 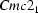

*a* = 15.501 (3) Å
*b* = 10.8569 (17) Å
*c* = 8.1579 (13) Å
*V* = 1372.9 (4) Å^3^
*Z* = 4
Mo *K*α radiation
μ = 0.20 mm^−1^
*T* = 100 K
0.60 × 0.15 × 0.13 mm

#### Data collection

Bruker APEXII CCD diffractometer
Absorption correction: multi-scan (*SADABS*; Bruker, 2005 ▶) *T*_min_ = 0.891, *T*_max_ = 0.975
5356 measured reflections
1317 independent reflections
1272 reflections with *I* > 2σ(*I*)
*R*_int_ = 0.031

#### Refinement

*R*[*F* ^2^ > 2σ(*F* ^2^)] = 0.028
*wR*(*F* ^2^) = 0.084
*S* = 1.04
1317 reflections
108 parameters
1 restraint
H atoms treated by a mixture of independent and constrained refinement
Δρ_max_ = 0.22 e Å^−3^
Δρ_min_ = −0.22 e Å^−3^
Absolute structure: Flack (1983 ▶), 604 Friedel pairs
Flack parameter: −0.11 (10)

### 

Data collection: *APEX2* (Bruker, 2005 ▶); cell refinement: *SAINT* (Bruker, 2005 ▶); data reduction: *SAINT*; program(s) used to solve structure: *SHELXS97* (Sheldrick, 2008 ▶); program(s) used to refine structure: *SHELXL97* (Sheldrick, 2008 ▶); molecular graphics: *Mercury* (Macrae *et al.*, 2008 ▶); software used to prepare material for publication: *SHELXTL* and *enCIFer* (Allen *et al.*, 2004 ▶).

## Supplementary Material

Crystal structure: contains datablock(s) I, global. DOI: 10.1107/S1600536811046058/hb6483sup1.cif

Structure factors: contains datablock(s) I. DOI: 10.1107/S1600536811046058/hb6483Isup2.hkl

Additional supplementary materials:  crystallographic information; 3D view; checkCIF report

## Figures and Tables

**Table 1:** Hydrogen-bond geometry (Å, °)

*D*—H⋯*A*	*D*—H	H⋯*A*	*D*⋯*A*	*D*—H⋯*A*
N1—H1*A*⋯O1^i^	0.88	2.22	2.982 (2)	145

Symmetry code: (i) 
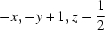
.

**Fig. 1.**
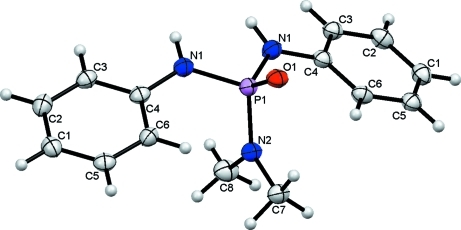
The molecular structure of the title compound with ellipsoids shown at the 50% probability level.

**Fig. 2.**
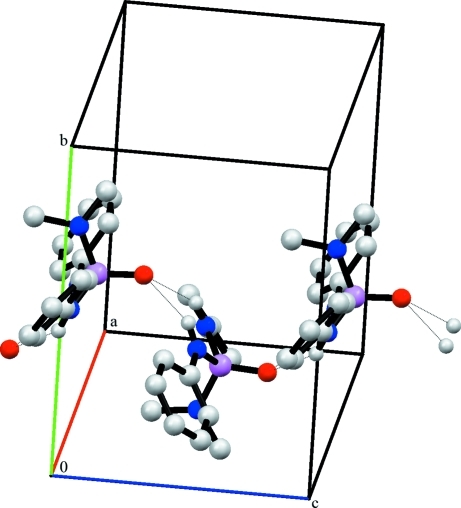
A view of the crystal packing showing the formation of 1-D arrangement through N—H···O hydrogen bonds (shown as dotted lines), carbon bound H atoms have been omitted for clarity.

## supplementary crystallographic information

### Comment

The title molecule, Fig. 1, has crystallographically imposed mirror symmetry. In the (CH_3_)_2_NP(O) unit, the O—P—N—C torsion angles, showing the orientations of the methyl groups with respect to the phosphoryl group, are 0.0 and 180.0°.

The P═O and P—N bond lengths and the C—N—P bond angles match those found for the other compounds having a [(N)P(O)(N)_2_] skeleton (Sabbaghi *et al.*, 2011).

The tetrahedral geometry of the phosphorus atom is significantly distorted as it has been noted for the other phosphoric triamides: the bond angles at the P atom vary in the range from 98.98 (11) [N1^i^—P1—N1; symmetry code (i): -x, y, z] to 115.28 (7)° [O1—P1—N1].

The O atom of the P═O group acts as a double hydrogen-bond acceptor (Pourayoubi *et al.*, 2011) to form the [N—H]_2_···O(P) grouping within a 1-D hydrogen-bonded arrangement along the *c* axis (Fig. 2, Table 1).

### Experimental

Synthesis of ((CH_3_)_2_N)P(O)Cl_2_: [(CH_3_)_2_NH_2_]Cl (0.184 mol) and P(O)Cl_3_ (0.552 mol) were refluxed for 8 h and afterwards the excess of P(O)Cl_3_ was removed in vacuum.

Synthesis of title compound: to a solution of ((CH_3_)_2_N)P(O)Cl_2_ (3.7 mmol) in CH_3_CN (15 ml), a solution of aniline (14.8 mmol) in CH_3_CN (25 ml) was added at 273 K. After 4 h stirring, the solvent was removed and product was washed with deionized water and recrystallized from CH_3_CN at room temperature to yield colourless rods.

### Refinement

The H4, H11, H12 and H13 were found in difference Fourier maps and refined with isotropic displacement parameters. The others hydrogen atom positions of the C-H and N-H units were calculated and refined in a riding-model with appropriate HFIX command in SHELXL-97.

### Crystal data

**Table:**

C_14_H_18_N_3_OP	*D*_x_ = 1.332 Mg m^−^^3^
*M**_r_* = 275.28	Melting point: NOT MEASURED K
Orthorhombic, *C**m**c*2_1_	Mo *K*α radiation, λ = 0.71073 Å
*a* = 15.501 (3) Å	Cell parameters from 3006 reflections
*b* = 10.8569 (17) Å	θ = 3.4–25.5°
*c* = 8.1579 (13) Å	µ = 0.20 mm^−^^1^
*V* = 1372.9 (4) Å^3^	*T* = 100 K
*Z* = 4	Rod, colourless
*F*(000) = 584	0.60 × 0.15 × 0.13 mm

### Data collection

**Table:**

Bruker APEXII CCD diffractometer	1317 independent reflections
Radiation source: fine-focus sealed tube	1272 reflections with *I* > 2σ(*I*)
graphite	*R*_int_ = 0.031
φ and ω scans	θ_max_ = 25.5°, θ_min_ = 2.3°
Absorption correction: multi-scan (*SADABS*; Bruker, 2005)	*h* = −18→14
*T*_min_ = 0.891, *T*_max_ = 0.975	*k* = −13→11
5356 measured reflections	*l* = −9→9

### Refinement

**Table:**

Refinement on *F*^2^	Secondary atom site location: difference Fourier map
Least-squares matrix: full	Hydrogen site location: inferred from neighbouring sites
*R*[*F*^2^ > 2σ(*F*^2^)] = 0.028	H atoms treated by a mixture of independent and constrained refinement
*wR*(*F*^2^) = 0.084	*w* = 1/[σ^2^(*F*_o_^2^) + (0.0623*P*)^2^] where *P* = (*F*_o_^2^ + 2*F*_c_^2^)/3
*S* = 1.04	(Δ/σ)_max_ = 0.001
1317 reflections	Δρ_max_ = 0.22 e Å^−^^3^
108 parameters	Δρ_min_ = −0.22 e Å^−^^3^
1 restraint	Absolute structure: Flack (1983), 604 Friedel pairs
Primary atom site location: structure-invariant direct methods	Flack parameter: −0.11 (10)

### Special details

**Table:**

Geometry. All e.s.d.'s (except the e.s.d. in the dihedral angle between two l.s. planes) are estimated using the full covariance matrix. The cell e.s.d.'s are taken into account individually in the estimation of e.s.d.'s in distances, angles and torsion angles; correlations between e.s.d.'s in cell parameters are only used when they are defined by crystal symmetry. An approximate (isotropic) treatment of cell e.s.d.'s is used for estimating e.s.d.'s involving l.s. planes.
Refinement. Refinement of *F*^2^ against ALL reflections. The weighted *R*-factor *wR* and goodness of fit *S* are based on *F*^2^, conventional *R*-factors *R* are based on *F*, with *F* set to zero for negative *F*^2^. The threshold expression of *F*^2^ > σ(*F*^2^) is used only for calculating *R*-factors(gt) *etc*. and is not relevant to the choice of reflections for refinement. *R*-factors based on *F*^2^ are statistically about twice as large as those based on *F*, and *R*- factors based on ALL data will be even larger.

### Fractional atomic coordinates and isotropic or equivalent isotropic displacement parameters (Å^2^)

**Table:**

	*x*	*y*	*z*	*U*_iso_*/*U*_eq_	
H13	−0.0531 (17)	0.819 (2)	−0.158 (3)	0.043 (6)*	
H11	0.0000	0.708 (4)	−0.186 (5)	0.052 (11)*	
H12	0.0000	0.849 (4)	0.262 (6)	0.053 (11)*	
H4	−0.0525 (13)	0.9247 (19)	0.112 (3)	0.028 (5)*	
P1	0.0000	0.62183 (5)	0.12667 (6)	0.02090 (19)	
O1	0.0000	0.63022 (15)	0.3089 (2)	0.0251 (4)	
N1	0.08103 (10)	0.54350 (14)	0.0464 (2)	0.0254 (4)	
H1A	0.0696	0.4706	0.0047	0.031*	
N2	0.0000	0.76094 (19)	0.0485 (3)	0.0243 (5)	
C1	0.34009 (13)	0.66067 (19)	0.0212 (2)	0.0271 (4)	
H1	0.3986	0.6864	0.0142	0.033*	
C2	0.31200 (13)	0.55744 (18)	−0.0645 (2)	0.0283 (4)	
H2	0.3515	0.5125	−0.1303	0.034*	
C3	0.22643 (11)	0.51958 (17)	−0.0546 (2)	0.0243 (4)	
H3	0.2080	0.4485	−0.1128	0.029*	
C4	0.16777 (13)	0.58505 (18)	0.0398 (2)	0.0232 (4)	
C5	0.28212 (11)	0.72536 (16)	0.1165 (3)	0.0252 (4)	
H5	0.3011	0.7954	0.1762	0.030*	
C6	0.19609 (10)	0.68909 (16)	0.1260 (3)	0.0241 (4)	
H6	0.1566	0.7349	0.1910	0.029*	
C7	0.0000	0.8737 (2)	0.1449 (4)	0.0269 (6)	
C8	0.0000	0.7773 (3)	−0.1284 (4)	0.0329 (6)	

### Atomic displacement parameters (Å^2^)

**Table:**

	*U*^11^	*U*^22^	*U*^33^	*U*^12^	*U*^13^	*U*^23^
P1	0.0224 (3)	0.0191 (3)	0.0212 (3)	0.000	0.000	0.0002 (3)
O1	0.0304 (10)	0.0227 (9)	0.0221 (10)	0.000	0.000	0.0036 (7)
N1	0.0246 (8)	0.0218 (8)	0.0300 (8)	−0.0009 (6)	0.0010 (6)	−0.0034 (6)
N2	0.0304 (11)	0.0218 (11)	0.0208 (12)	0.000	0.000	−0.0001 (8)
C1	0.0239 (9)	0.0308 (10)	0.0265 (9)	0.0005 (8)	−0.0006 (8)	0.0058 (8)
C2	0.0286 (9)	0.0314 (10)	0.0248 (9)	0.0073 (8)	0.0025 (8)	0.0006 (8)
C3	0.0289 (9)	0.0215 (9)	0.0225 (9)	0.0025 (7)	−0.0021 (8)	0.0006 (7)
C4	0.0264 (9)	0.0208 (10)	0.0224 (10)	0.0021 (8)	−0.0024 (7)	0.0032 (7)
C5	0.0296 (8)	0.0230 (8)	0.0230 (9)	0.0005 (7)	−0.0033 (9)	0.0038 (8)
C6	0.0269 (8)	0.0226 (8)	0.0227 (7)	0.0043 (7)	0.0013 (8)	−0.0007 (8)
C7	0.0342 (13)	0.0224 (13)	0.0241 (14)	0.000	0.000	0.0001 (10)
C8	0.0446 (17)	0.0310 (16)	0.0232 (14)	0.000	0.000	0.0029 (12)

### Geometric parameters (Å, °)

**Table:**

P1—O1	1.489 (2)	C2—H2	0.9500
P1—N2	1.639 (2)	C3—C4	1.388 (3)
P1—N1^i^	1.6521 (16)	C3—H3	0.9500
P1—N1	1.6522 (16)	C4—C6	1.401 (3)
N1—C4	1.419 (2)	C5—C6	1.393 (2)
N1—H1A	0.8800	C5—H5	0.9500
N2—C8	1.454 (3)	C6—H6	0.9500
N2—C7	1.455 (3)	C7—H12	0.99 (5)
C1—C5	1.380 (3)	C7—H4	1.02 (2)
C1—C2	1.391 (3)	C8—H13	0.97 (3)
C1—H1	0.9500	C8—H11	0.89 (4)
C2—C3	1.391 (3)		
			
O1—P1—N2	109.39 (11)	C4—C3—C2	120.39 (18)
O1—P1—N1^i^	115.28 (7)	C4—C3—H3	119.8
N2—P1—N1^i^	108.66 (8)	C2—C3—H3	119.8
O1—P1—N1	115.28 (7)	C3—C4—C6	119.10 (17)
N2—P1—N1	108.66 (8)	C3—C4—N1	118.62 (16)
N1^i^—P1—N1	98.98 (11)	C6—C4—N1	122.28 (16)
C4—N1—P1	124.87 (13)	C1—C5—C6	120.74 (18)
C4—N1—H1A	117.6	C1—C5—H5	119.6
P1—N1—H1A	117.6	C6—C5—H5	119.6
C8—N2—C7	115.7 (2)	C5—C6—C4	119.99 (17)
C8—N2—P1	119.9 (2)	C5—C6—H6	120.0
C7—N2—P1	124.38 (19)	C4—C6—H6	120.0
C5—C1—C2	119.30 (18)	N2—C7—H12	107 (2)
C5—C1—H1	120.4	N2—C7—H4	108.2 (13)
C2—C1—H1	120.4	H12—C7—H4	113.7 (18)
C1—C2—C3	120.49 (18)	N2—C8—H13	107.6 (15)
C1—C2—H2	119.8	N2—C8—H11	115 (3)
C3—C2—H2	119.8	H13—C8—H11	105.1 (19)
			
O1—P1—N1—C4	−72.29 (16)	C1—C2—C3—C4	0.6 (3)
N2—P1—N1—C4	50.87 (18)	C2—C3—C4—C6	−0.5 (3)
N1^i^—P1—N1—C4	164.16 (11)	C2—C3—C4—N1	179.88 (17)
O1—P1—N2—C8	180.0	P1—N1—C4—C3	−169.43 (13)
N1^i^—P1—N2—C8	−53.37 (7)	P1—N1—C4—C6	11.0 (2)
N1—P1—N2—C8	53.37 (7)	C2—C1—C5—C6	−0.7 (3)
O1—P1—N2—C7	0.0	C1—C5—C6—C4	0.7 (3)
N1^i^—P1—N2—C7	126.63 (7)	C3—C4—C6—C5	−0.1 (3)
N1—P1—N2—C7	−126.63 (7)	N1—C4—C6—C5	179.45 (18)
C5—C1—C2—C3	0.0 (3)		

Symmetry codes: (i) −*x*, *y*, *z*.

### Hydrogen-bond geometry (Å, °)

**Table:**

*D*—H···*A*	*D*—H	H···*A*	*D*···*A*	*D*—H···*A*
N1—H1A···O1^ii^	0.88	2.22	2.982 (2)	145

Symmetry codes: (ii) −*x*, −*y*+1, *z*−1/2.
